# Gamma Knife Radiosurgery as a Salvage Treatment for Nasopharyngeal Carcinoma with Skull Base and Intracranial Invasion (T4b)

**DOI:** 10.3390/life12111880

**Published:** 2022-11-14

**Authors:** Shao-Ang Chu, Tai-Been Chen, Han-Jung Chen, Kuo-Wei Wang, Jui-Sheng Chen, Fu-Cheng Chuang, Hao-Kuang Wang, Cheng-Loong Liang

**Affiliations:** 1 School of Medicine, I-Shou University, Kaohsiung 82445, Taiwan; 2 Department of Neurosurgery, E-Da Hospital, I-Shou University, Kaohsiung 82445, Taiwan; 3 Department of Information Engineering, I-Shou University, Kaohsiung 84001, Taiwan; 4 Department of Medical Imaging and Radiological Sciences, I-Shou University, Kaohsiung 82445, Taiwan; 5 Institute of Statistics, National Yang Ming Chiao Tung Univsersity, Hsinchu 300093, Taiwan; 6 Department of Neurosurgery, E-Da Cancer Hospital, I-Shou University, Kaohsiung 82445, Taiwan; 7 Department of Radiation Oncology, E-Da Hospital, I-Shou University, Kaohsiung 82445, Taiwan

**Keywords:** nasopharyngeal carcinoma, salvage therapy, prognostic factors, TNM staging, brain imaging

## Abstract

It is usually difficult to achieve good outcomes with salvage treatment for recurrent nasopharyngeal carcinoma (NPC) because of its deep-seated location, surrounding critical structures, and patient history of high-dose irradiation. Gamma Knife radiosurgery (GKS) is a treatment option for malignancies with skull base and intracranial invasion. We conducted a retrospective, observational, single-center study including 15 patients with recurrent NPC (stage T4b) involving the skull base and intracranial invasion, who underwent GKS as a salvage treatment. Patients were enrolled over 12 years. Per a previous study, the TNM classification T4b was subclassified into T4b1 and T4b2, defined as the involvement of the skull base or cavernous sinus with an intracranial extension of <5 mm and >5 mm, respectively. The effect of prognostic factors, including age, sex, survival period, magnetic resonance imaging (MRI) presentation, presence of other distant metastases, tumor volume, marginal dose, maximal dose, and Karnofsky Performance Status (KPS), on outcomes was analyzed. The patients with T4b1 NPC (*p* = 0.041), small tumor volume (*p* = 0.012), higher KPS (*p* < 0.001), and no other metastasis (*p* = 0.007) had better outcomes after GKS treatment, suggesting that it is a viable treatment modality for NPC. We also suggest that detailed brain imaging studies may enable the early detection of intracranial invasion.

## 1. Introduction

Carcinoma of the nasopharynx (NPC), arising from the narrow tubular passage behind the nasal cavity, is a rare tumor that affects approximately 0.5–2 in every 100,000 people in the United States and Western Europe [1]. Histologically, NPC is predominantly an undifferentiated, nonkeratinizing squamous cell carcinoma [2]. However, the racial and geographic distributions of this disease are remarkable. A marked elevation of NPC incidence is seen in Southern Asia, including Taiwan (5–10 in every 100,000 people). As it is a radiosensitive tumor and its anatomical location limits a surgical approach, radiotherapy remains the mainstay in NPC treatment. Primary NPC is commonly treated with relatively high-dose radiotherapy (66–70 Gy) using either external beam radiation therapy (RT) alone or in combination with chemotherapy [3,4].

For patients with early stage I or II of the disease, RT with or without chemotherapy can achieve excellent locoregional control and prevent distant metastases, as shown in randomized trials [5,6]. Treatment options for an advanced-stage of the disease include induction chemotherapy followed by concurrent chemoradiation or concurrent chemoradiation with or without adjuvant chemotherapy [7]. Even with radical radiotherapy, approximately 7–15% of patients remain at risk of persistent or recurrent disease, and 10–40% of patients experience NPC recurrence within 1–2 years after initial treatment [8,9]. Treatment of recurrent and metastatic NPC remains challenging: both conditions are the most common causes of death among patients with NPC, with a median overall survival of 10–36 months [10]. Unfortunately, retreatment using conventional techniques is associated with poor outcomes and/or a high incidence of late complications. Salvage treatment for recurrent NPC is usually arduous because of the deep-seated location of NPC, along with the surrounding critical structures. For the treatment of small (i.e., cT1 to T2) recurrent local lesions eligible for resection, salvage operations with endoscopic nasopharyngectomy should be performed rather than reirradiation with or without concurrent chemotherapy because this approach improved the overall survival and decreased long-term toxicity in a phase III trial [11]. However, salvage operations with endoscopic nasopharyngectomy are technically challenging because of the limited access to the nasopharynx. For larger (T3 to T4) locoregionally recurrent nasopharyngeal tumors or those that are unresectable, including cases of the invasion of the skull base region, reirradiation still serves as a treatment alternative. Reirradiation presents a therapeutic challenge because the safe dose is limited by previous radiotherapy treatments and the tolerance of healthy tissues; significant acute and late toxicities must be expected [12]. Further, radioresistance induced by previous therapy poses a major problem, particularly in patients who develop recurrence after initial high radiation doses administered as primary therapy along with chemotherapy. A significant proportion of patients with local failure can still be successfully salvaged and can achieve long-term survival; therefore, aggressive treatment should be considered whenever possible [13].

Stereotactic radiosurgery (SRS) has recently evolved as a treatment option for malignant skull base and intracranial lesions, and several reports have suggested that the treatment is effective in salvaging skull base invasion in NPC, although complications were also observed in some patients after treatment [14,15,16]. The dose distribution of stereotactic radiosurgery provides better homogeneity, especially lesions which involve areas distant to the nasopharyngeal mucosa [17]. Information regarding SRS for salvaging patients with late-stage relapse is still scarce, and the effectivity of SRS, compared with other salvage treatments, remains unknown. Only a few studies have reported the use of SRS in salvaging late-stage tumor relapse in NPC [18,19].

Gamma Knife radiosurgery (GKS) is a very precise form of SRS that focuses on intense beams of gamma rays with pinpoint accuracy to treat lesions in the brain and has recently evolved as an alternative treatment for malignancy with skull base and intracranial invasion [20]. Previous studies suggested that GKS is an acceptable salvage treatment option for patients with recurrent NPC who have previously received RT [21,22]. Management of the skull base and intracranial invasion (T4) in patients with NPC who have previously received full-dose (brain stem, 54–56 Gy) radiotherapy remains an important issue, and few studies have used GKS to deal with these conditions [21]. Therefore, this study aimed to investigate the feasibility of GKS for intracranial invasion in patients with NPC who had already received radiotherapy.

## 2. Materials and Methods

### 2.1. Patients

The present study was designed as an observational and retrospective cohort study with a longitudinal follow-up. This single-center study included patients with recurrent NPC involving the skull base and intracranial invasion (T4b) [23]. It was approved by the institutional review board (EMRP05110N). The study inclusion criteria were patients with image-based and histologically confirmed diagnosis of NPC stage T4b, those with tumor recurrence who underwent GKS, and patients for whom at least three months of clinical and neuroimaging follow-up data were available. Exclusion criteria included patients lost to clinical and/or radiological follow-up. Patients were divided into the good outcome (survival for ≥2 years after GKS) and poor outcome (survival <2 years after GKS) groups.

The American Joint Committee on Cancer TNM staging system was used for staging of patients with NPC [24]. The T4 stage in the TNM staging system was further classified into T4a and T4b according to the site of invasion, as previously reported [23]; primary nasopharyngeal tumors involving the masticator space only were staged as T4a, while those involving the intracranial region, cranial nerves, and/or the orbit were staged as T4b.

### 2.2. Magnetic Resonance Imaging Staging of T4b1 and T4b2

**Figure 1:**
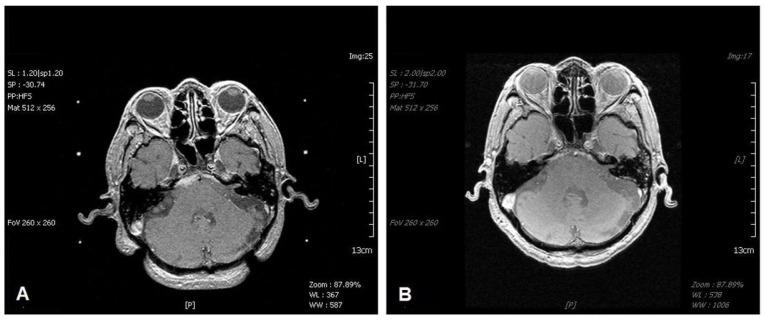
T1-weighted magnetic resonance imaging (MRI) of the brain revealing a well-enhanced tumor with extension to the right cavernous sinus and minimal intradural invasion (T4b1) after gadolinium injection (**A**). The tumor was treated with GKS using a margin dose of 12 Gy. Six months later, the follow-up brain MRI reveals tumor regression (**B**).

**Figure 2:**
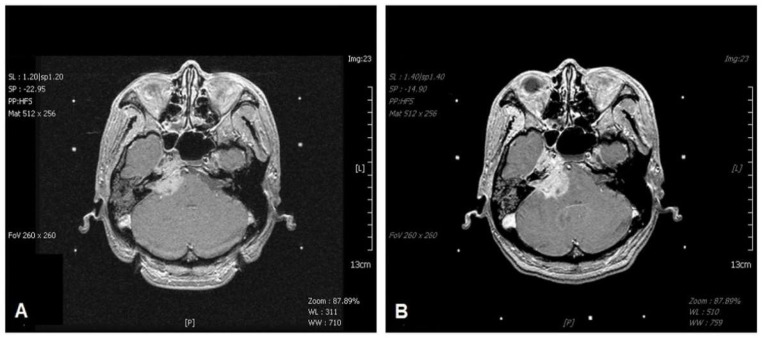
T1-weighted magnetic resonance imaging (MRI) of the brain revealing a well-enhanced tumor with extension to the right cavernous sinus and intradural invasion (T4b2) after gadolinium injection (**A**). The tumor was treated with GKS using a margin dose of 12 Gy. Six months later, the follow-up brain MRI reveals tumor progression (**B**).

All patients in the study group were classified as having stage T4b tumors [23]. We further subdivided T4b tumors into two groups (T4b1 vs. T4b2) according to the extent of intracranial invasion. T4b1 was defined as the involvement of the skull base or cavernous sinus with minimal intracranial extension (<5 mm, Figure 1), while T4b2 was defined as significant involvement of the intracranial region (≥5 mm, Figure 2).

### 2.3. Clinical Data Collection

The clinical data collected included age and performance status (Karnofsky Performance Status (KPS)) at GKS, cranial nerve deficits, magnetic resonance imaging (MRI) stage, history of pre-SRS radiotherapy, other metastases, overall survival, and tumor progression or regression. The collected GKS parameters included tumor volume, marginal dose, number of isocenters, prescription isodose line, and maximal dose.

### 2.4. Gamma Knife Radiosurgery Technique

The radiosurgical techniques used herein have been previously described [25,26,27]. GKS was performed following administration of a local anesthetic agent. After a Leksell model G stereotactic frame (Elekta AB) was affixed to the head, each patient underwent stereotactic MRI to identify the tumor. High-resolution, thin-slice (1 mm), post-contrast T1- and T2-weighted brain MRI scans were obtained and used for stereotactic targeting. Targeting was based on axial images. These imaging sequences provide a graphic depiction of the optic nerve, brain stem, and tumor. Treatment planning was performed using the Leksell GammaPlan software (version 5.32) for the C Gamma Knife (Elekta AB) model. The target volumes were drawn in the presence of a neuroradiologist, neurosurgeon, and physicist. Each treatment plan was created using the same clinical criteria: a plan that was as conformal as possible without exceeding the dose constraints for critical structures. The plug function was used to protect the critical structures.

### 2.5. Statistical Analysis

In this study, data are presented as means and standard deviation for continuous variables and as frequencies and percentages for categorical variables. The chi-square test with Fisher’s correction was utilized to examine the relationship between categorical predictors (i.e., sex, brain MRI, and other metastases) and binary response variables (good or poor outcome). Statistics involving means were calculated using an unpaired Student’s *t*-test. The cut-off values of predictor variables were determined using the Youden index after receiver operating characteristic (ROC) analysis. Logistic regression was not applicable due to the small number of cases. Meanwhile, the Kaplan–Meier Plot was applied to compare the survival rates for KPS which was categorized two groups. All tests were two-tailed, and a *p*-value of <0.05 was considered statistically significant. Data analyses were performed using SPSS version 24 (IBM SPSS Inc., Chicago, IL, USA).

## 3. Results

### 3.1. Characteristics of the Patients

From January 2006 to December 2017, 15 patients with recurrent NPC involving the skull base and intracranial invasion (stage T4b) were enrolled in this study. All patients had received conventional irradiation and chemotherapy for the original NPC lesions and the recurrent tumors. Patient characteristic data are summarized in Table 1. Several prognostic factors were evaluated in our study to determine their prognostic impact on patients’ survival in the two groups (good and poor outcomes), including age, sex, survival period, MRI presentation, presence of other metastases, tumor volume, number of isocenters, prescription isodose line, marginal dose, maximal dose, and KPS. No new development of cranial nerve deficits was observed in our patients.

### 3.2. Prognostic Significance of Tumor Volume, MRI Staging, KPS, and Other Metastases

The significant predictors of good outcomes included small tumor volume (*p* = 0.012), high KPS (*p* < 0.001), MRI stage T4b1 (*p* = 0.041), and absence of systemic metastasis (*p* = 0.007). The mean survival duration of patients with NPC T4b1 and T4b2 was 51.7 ± 30.0 months and 5.3 ± 2.4 months, respectively (*p* = 0.006) (Table 1).

### 3.3. ROC Analysis

**Figure 3:**
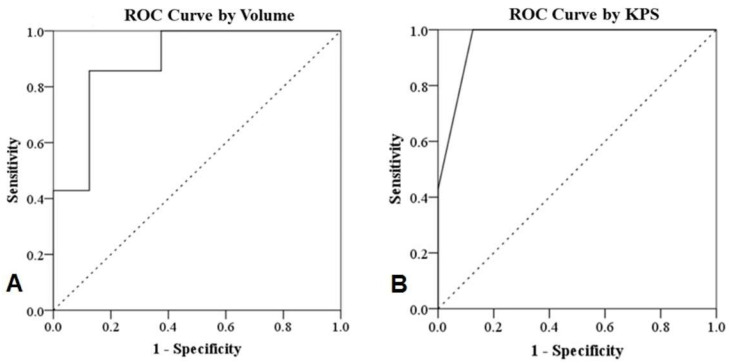
Receiver operating characteristic (ROC) curves of tumor volume (**A**) and KPS (**B**) after GKS in patients with T4b NPC. KPS: Karnofsky Performance Status, GKS: Gamma Knife radiosurgery.

The details of the ROC analysis are shown in Figure 3 and Table 2. The cut-off values of the variables were determined using the Youden index after ROC analysis. A tumor volume of <11.75 cm^3^ (*p* = 0.011) and KPS of >85 (*p* = 0.003) were predictive of good patient outcomes.

### 3.4. The Kaplan–Meier Plot for KPS

**Figure 4:**
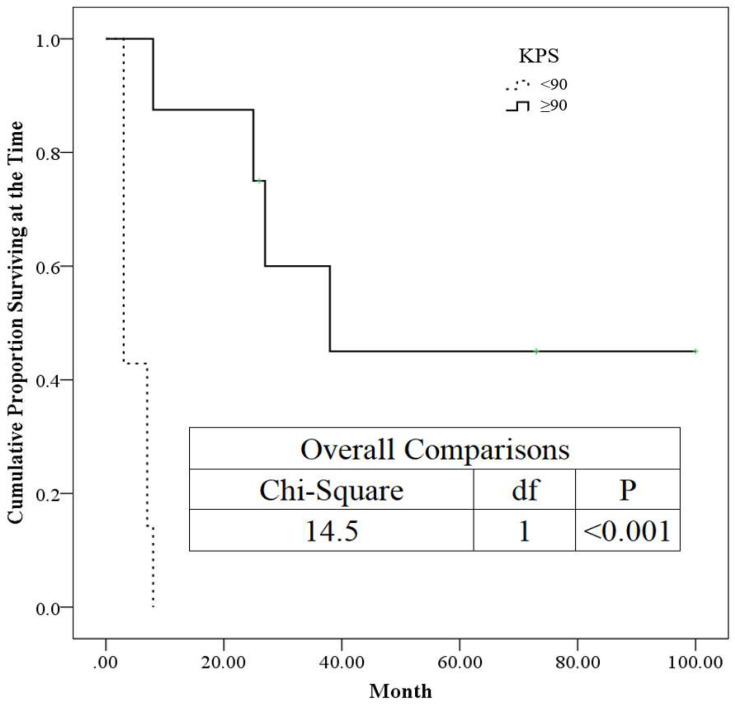
The Kaplan–Meier plot for categorical KPS.

In this study, the KPS was categorized as <90 and ≥90. The Kaplan–Meier plot was generated by using categorized KPS to consider them as time-to-event data. Meanwhile, the *p*-values (<0.001) smaller than 0.05 were demonstrated as statistically significance in this work. Hence, the higher the KPS obtained, the better survival rate is achieved (Figure 4).

## 4. Discussion

Even though survival and local control rates are high after appropriate treatment for early and intermediate-stage NPC, long-term local nasopharyngeal control is obtained for only 40–70% of T3 and 40–50% of T4 lesions [8,9]. Local recurrence after external beam RT remains a major cause of treatment failure, and isolated local relapse is the major recurrent pattern. Moreover, recurrence and metastasis are the most common causes of death [10,28]. Prolonged survival is achievable in patients who are carefully selected and appropriately treated, and aggressive local salvage therapy can help achieve a better survival rate [13]. Routine detailed MRI studies of the head and neck to assess locoregional disease prior to the initial treatment of the original NPC may be recommended based on our findings. Eight and seven patients with NPC had minimal (T4b1) and extended (T4b2) intracranial invasion, respectively. Staging in all cases was performed based on detailed enhanced MRIs, which may suggest that the small extension of primary lesions may have been overlooked on the initial imaging. Therefore, precise imaging studies could facilitate the early detection of intracranial invasion and decrease the rate of undetected extended lesions or the under-evaluation of the primary disease stage.

For patients with small local recurrences and no distant metastases, resection of such lesions could be effective if the recurrence does not involve the bone or cranial nerves. Surgery in this setting is technically challenging, and the goal is to achieve an adequate margin while preserving the neurovascular bundle and restoring the critical mucosal barriers [11]. However, most nasopharyngeal recurrent lesions are more extensive, and reirradiation at relatively high doses is often necessary [12]. Successful salvage or palliation of NPC recurrence is challenging owing to multiple factors. The complexity of the skull base anatomy often precludes complete surgical resection or the optimal placement of a radioactive implant. However, a recent study demonstrated three-year survival rates as high as 85.8% after salvage endoscopic nasopharyngectomy in patients with locally recurrent NPC confined to the nasopharyngeal cavity, post-naris or nasal septum, superficial parapharyngeal space, or base wall of the sphenoid sinus [11]. A poor prognosis is associated with a higher T classification of recurrent tumors (involvement of the skull base, cranial nerve, dura, or brain) and the development of severe adverse effects [12]. With low response rates, the efficacy of chemotherapy after recurrence is low, and approximately half of the patients with apparent local recurrence have synchronous distant metastases [28]. Furthermore, a recent study revealed that a worse prognosis was associated with an elevated level of the Epstein–Barr virus (EBV)-DNA, and an EBV-DNA load of more than 70 copies/cell was associated with a better outcome in terms of 7-year disease-free survival [29].

Multiple modalities exist for the salvage treatment of local recurrences of NPC. SRS is an effective therapy for the palliative treatment of patients with minimal treatment-related morbidity [30,31]. The main advantage of SRS is a rapid dose decrease around the target volume, which spares sensitive adjacent vital structures and previously irradiated tissue. GKS, a very precise form of SRS, focuses intense gamma ray beams with pinpoint accuracy to treat lesions in the brain, thereby providing better dose homogeneity. It has evolved as an alternative treatment option for malignancies with skull base and intracranial invasion [20].

Fifteen patients with recurrent NPC involving skull base and intracranial invasion were enrolled in our study. There is no standardized marginal prescription dose for recurrent tumors; a dose of at least 12 Gy is recommended based on previous scientific articles and our experience [21]. We ensured that radiation doses to the optic nerve, optic chiasm, and brain stem were <8 Gy when patients had no tumor involvement within these structures.

We subdivided the T4b classification into two grades: T4b1, defined as involvement of the skull base or cavernous sinus with minimal intracranial extension (<5 mm), and T4b2, defined as the involvement of the intracranial region (≥5 mm). Table 1 presents the prognostic factors analyzed in our study to determine their effect on patient survival in the two groups. We found that significant predictors of good outcomes included small tumor volume (*p* = 0.012), high KPS (*p* < 0.001), negative other systemic metastasis (*p* = 0.007), and the T4b1 stage (*p* = 0.041) in MRI performed before treatment with GKS. The findings regarding small tumor volume and negative systemic metastasis were also reported in a previous study [32]. Table 2 presents the results of the ROC analysis, which revealed that a KPS score of >85 and tumor volume <11.75 cm^3^ can predict a good outcome for patients; this result is consistent with the findings presented in Table 1. Furthermore, a KPS no less than 90 is associated with 50% probability for survival over 40 months, as shown in Figure 4. A recent study involving fractionated robotic stereotactic body radiosurgery administered as an optional salvage treatment for the reirradiation of locally recurrent nasopharyngeal carcinoma demonstrated that the prognostic factors for local failure-free survival were a cumulative total radiotherapy dose, gross tumor volume, and recurrence time interval, and that treatment-related mortality was vascular in nature [33]. These findings support ours and demonstrate that SRS is a promising treatment modality for recurrent NPC. Figure 1 shows the viable treatment options for T4b1 lesions via GKS.

NPC involving the intracranial region always invades the skull base and neural foramina. Liu et al. reported that intracranial and orbital extensions are frequently associated with MRI-detected intracranial nerve involvement, including an invasion of the cavernous sinus segment of the cranial nerves III and VI, trigeminal ganglion, cranial nerves in the cistern, inferior orbital fissure, orbital apex, and superior orbital fissure [34]. Malignancies involving MRI-detected cranial nerve involvement have a high distant metastasis rate and a poor survival rate [34]. Batsakis et al. reported that carcinomas proliferate along the nerves within the lymphatic system of the epineurium and perineural sheaths [35]. Perineural invasion is a key clinicopathological feature in such cases, as it has been established as an adverse prognostic factor in many malignancies, including head and neck, colon and rectum, and prostate cancers. The same rule applies to NPC, wherein involvement of the cranial nerve is also a poor prognostic factor [36].

Our results demonstrate that the overall survival associated with T4b1 was better than that of the T4b2 lesions. It is presumed that tumor proliferation within the lymphatic system may increase the risk of distant metastasis, which could explain why patients with T4b2 NPC were more likely to experience relapse at distant sites. In contrast, the venous plexus is rich in the intracranial region, especially the cavernous sinus, and is conventionally thought to be a potential route of hematogenous dissemination for recurrent NPC, which is probably another reason for the poor survival rate of T4b2 patients. These results support the necessity of the T4b1/T4b2 subclassifications.

This study has several limitations. First, it was a retrospective, observational, single-center study. However, the standard salvage treatment for the recurrent high T stage has not yet been established, and patients enrolled in our study were mainly from the oncology department who had no other better treatment options. Second, the number of patients was relatively small, and the incidence of NPC is uncommon. Particularly in terms of the inclusion criteria of this study, recurrent disease with invasion of the skull base and cranium has rarely been reported. Therefore, further investigation would be indispensable.

## 5. Conclusions

GKS is a reasonable treatment modality for selected NPC patients with skull base recurrence, particularly in patients who are inoperable, have limited intracranial extension, a smaller tumor volume, a higher KPS, and no distal metastasis. Furthermore, our preliminary results suggest that GKS provides favorable tumor control with few complications in patients with T4b1 NPC. Detailed brain imaging studies prior to initial treatment for NPC may facilitate the early detection of intracranial invasion. However, our findings should be validated, in terms of long-term outcomes and late complications, in future studies.

## Figures and Tables

**Table 1:** Comparison of the clinical characteristics of patients between group 1 (good outcome) and group 2 (poor outcome).

Predictors	All (N = 15)	Group 1 (Good Outcome) (N = 7)	Group 2 (Poor Outcome) (N = 8)	*p*-Value
Gender (Male) (%)	12 (80)	5 (71)	7 (88)	0.569 ^†^
Age (Mean ± SD) (year)	51.3 ± 10.3	56.4 ± 10.8	46.8 ± 7.9	0.76
Prescription isodose line	51.3 ± 1.3	50.7 ± 1.9	50.0 ± 0	0.231
Number of isocenters	21.7 ± 6.4	19.6 ± 6.0	23.6 ± 6.5	0.302
Tumor volume (Mean ± SD) (cm^3^)	12.0 ± 6.2	7.9 ± 4.1	15.5 ± 5.7	0.012
Marginal dose (Mean ± SD) (Gy)	12.0 ± 2.0	12.2 ± 1.5	11.8 ± 2.4	0.659
Maximal dose (Mean ± SD) (Gy)	22.9 ± 4.0	22.9 ± 2.7	23.0 ± 5.0	0.956
KPS (Mean (Median) ± SD)	86.0 (90.0) ± 9.9	94.3 (90.0) ± 5.3	78.8 (80.0) ± 6.4	<0.001
Brain MRI				0.041 ^†^
Extradural (T4b1)	8	6	2	
Intradural (T4b2)	7	1	6	
Other metastasis				0.007 ^†^
No	9	7	2	
Yes	6	0	6	
Survival (Mean ± SD) (months)	26.9 ± 31.1	51.7 ± 30.0	5.3 ± 2.4	0.006

Note: ^†^ indicates using Fisher’s exact test.

**Table 2:** Diagnostic properties of different thresholds of tumor volume and KPS for predicting the outcome of patients with T4b nasopharyngeal carcinoma.

Variable	AUC	95% Confidence Interval		*p*-Value	Maximal Yuden Index	Cut-Off * Value
		Lower	Upper			
Volume	0.893	0.725	1.000	0.011	0.536	11.750
KPS	0.964	0.873	1.000	0.003	0.875	85.000

* According to the Youden index. AUC: Area under curve.

## Data Availability

The data presented in this study are available upon request from the corresponding author. The data are not publicly available due to restrictions, e.g., privacy and ethical concerns.
